# Influenza Vaccination Intention Among Caregivers in the Context of Highly Publicized Influenza Events: A Cross-Sectional Survey of Caregivers of Kindergarten and Primary School Children in Zhejiang, China

**DOI:** 10.3390/vaccines14050377

**Published:** 2026-04-23

**Authors:** Zhaokai He, Minchao Li, Yun Zeng, Rui Zhang, Jing Tao, Yumeng Wu, Jianwu Li, Guiwei Zhu, Qianhui Zheng, Junqi Yang, Liangliang Huo, Jing Wang

**Affiliations:** 1 Hangzhou Center for Disease Control and Prevention (Hangzhou Health Supervision Institution), Hangzhou 310021, China; hezhaokai@hzcdc.com.cn; 2 The Centre for Disease Control and Prevention of Haining County, Jiaxing 314400, China; 3 Hangzhou Qiantang District Center for Disease Control and Prevention (Hangzhou Qiantang District Health Inspection Institute), Hangzhou 310020, China; 4 Jiaojiang Center for Disease Control and Prevention and Jiaojiang Institute of Health Supervision, Taizhou 318000, China; 5 Cixi Center for Disease Control and Prevention, Ningbo 315300, China; 6 Jiashan County Center for Disease Control and Prevention, Jiaxing 314100, China; 7 Xianju County Center for Disease Control and Prevention, Taizhou 317300, China; 8 Tiantai County Center for Disease Control and Prevention, Taizhou 317200, China; 9 Kaihua County Center for Disease Control and Prevention, Quzhou 324300, China; 10 Wenzhou Center for Disease Control and Prevention (Wenzhou Health Supervision Institute), Wenzhou 315000, China

**Keywords:** influenza vaccine, vaccination intention, caregiver, children, cross-sectional survey

## Abstract

**Objective**: This study assessed the influence of a highly publicized influenza-related death event on caregivers’ influenza vaccination intention for kindergarten and primary school children in Zhejiang, China, and identified associated factors. **Methods**: A cross-sectional survey was conducted from March to April 2025 using a multi-stage, stratified cluster sampling method across 10 districts/counties. Caregivers completed electronic questionnaires covering sociodemographics, event awareness, vaccination history, hesitancy, and cognitive attitudes. Factors associated with vaccination intention were analyzed using chi-square tests and logistic regression. **Results**: Among 2153 caregivers, overall vaccination intention for the 2025 season was 60.10%, markedly higher than the 2024 season’s actual rate (27.45%). Caregiver awareness of this event was 91.92%, primarily via social media (92.02%). In univariate analyses, event-related characteristics were significantly associated with vaccination intention: perceived “completely objective” coverage showed the highest willingness (79.68%, χ^2^ = 79.92, *p* < 0.001), whereas the “exaggerated risk” (52.44%) and “unaware” (51.15%) groups showed lower willingness. Exposure frequency also correlated positively: low exposure (0–2 times) had 53.39% willingness, moderate (3–5) 61.11%, and high (≥6) 66.10% (χ^2^ = 27.75, *p* < 0.001). However, stronger vaccination intention was independently associated with factors such as no prior vaccination refusal [aOR(95% CI) = 2.74(2.03,3.72)] or hesitancy history [1.47(1.13,1.92)], greater information need (aOR = 6.42–8.83), and disbelief in influenza’s spontaneous resolution [1.39(1.08,1.77)]. Weaker intention was associated with poorer child health status [0.19(0.04,0.74)], no influenza vaccination in 2024 [0.41(0.30,0.55)], no influenza illness in 2024 [0.73(0.56,0.95)], belief in vaccine protection [0.60(0.46,0.79)], and the perception that most parents have their children vaccinated [0.70(0.53,0.93)]. **Conclusions**: Following a highly publicized celebrity influenza death, vaccination intention was primarily driven by caregivers’ cognitive, psychological, and behavioral experience factors. Caregivers who perceived event coverage as completely objective showed higher vaccination intention, while prior vaccination behavior exhibited inertia. Targeted interventions should enhance information credibility and focus on previously unvaccinated and vaccine-hesitant groups to improve coverage.

## 1. Introduction

Influenza is an acute respiratory infectious disease caused by influenza viruses, representing a substantial disease burden and public health challenge globally. According to World Health Organization estimates, influenza causes approximately 3 to 5 million severe cases and 290,000 to 650,000 respiratory-related deaths worldwide annually [1]. Children represent a high-risk population for influenza virus transmission due to their incompletely developed immune systems, congregate learning environments, and inadequately established personal hygiene practices. Domestic and international research has demonstrated that schools are high-incidence settings for influenza outbreaks. Data from China’s 2017–2018 surveillance year indicated that influenza-like illness outbreaks predominantly occurred in primary schools, secondary schools, and childcare institutions, accounting for 93.45% of all outbreaks [2]. International studies have demonstrated that extending school closure periods during influenza epidemics could reduce cases by 13–17%, with reductions of 18–23% among children; during peak influenza periods, case reductions could reach 39–45%, with reductions of 47–52% among children [3].

Influenza vaccination remains one of the most cost-effective measures for preventing influenza and its complications. Childhood vaccination not only reduces individual disease risk, but also plays a critical role in establishing school-based immunization barriers and controlling epidemic transmission. Multiple studies have confirmed that vaccinating primary and secondary school students against influenza can reduce school-based influenza outbreak risk by 50% to 90% [4,5]. Because the influenza vaccine is classified as a non-immunization schedule vaccine in China, vaccination uptake largely depends on parental autonomous choice and willingness to pay. Despite widespread international recommendations for vaccination and the World Health Organization’s designation of children as a priority population for influenza vaccination [6], childhood influenza vaccination rates in China remain substantially below international levels [7,8]. During the 2021–2022 influenza season, the overall population vaccination rate was only 2.5%, while the rate among children aged 6 months to 14 years was 12.6% [9,10]. The low vaccination rate among Chinese children primarily stems from parental vaccine hesitancy, with approximately 40% of parents of children under 5 years expressing hesitancy toward influenza vaccination [11,12], citing reasons such as insufficient vaccine knowledge and concerns about safety and efficacy [13].

In February 2025, a prominent public figure from China’s Taiwan region died in Japan from influenza complicated by pneumonia. This event—the death of celebrity due to influenza—rapidly ascended to the top of trending searches on the Chinese social media platform Weibo, accumulating over 1.5 billion views, with the total readership across related topics exceeding 3 billion. These figures reflects the substantial impact of the event within Chinese-speaking communities worldwide [14]. The incident not only captured public attention regarding influenza, but also prompted a critical re-evaluation of its potential health threats, particularly for individuals with pre-existing medical conditions. Against this backdrop, the widespread media coverage of this highly publicized influenza-related death amplified public risk perception regarding the disease, creating a unique natural experiment for studying the impact of public health emergencies on individual health decision-making. Such focal events may influence preventive health behaviors by altering public risk perception and health beliefs. Similar research has demonstrated that celebrity health events can increase the utilization of related health services or vaccination consultations [15,16].

However, evidence regarding the impact of such highly publicized influenza death events on childhood vaccination intention remains limited. Existing studies have primarily focused on routine health education, policy advocacy, or general determinants of parental vaccine hesitancy, with relatively limited evidence on how sudden, negative public health events—amplified through extensive media coverage—reshapes parental vaccination intentions for children. This research gap highlights the relevance of the present study: given the low childhood influenza vaccination rate in China and the potential influence of high-profile public health events on health decision-making, exploring the impact of this specific event on parental vaccination intention is crucial for developing targeted strategies to improve childhood influenza vaccination coverage. Therefore, this study aimed to investigate influenza vaccination intention and its associated factors among caregivers of children in Zhejiang Province, China, in the context of this highly publicized celebrity influenza-related death event, and examined how event-related, behavioral, and cognitive factors were associated with willingness to vaccinate.

## 2. Materials and Methods

### 2.1. Study Population

This cross-sectional survey was conducted in Zhejiang Province from March to April 2025. The study population comprised caregivers of children aged 3 to 12 years. Given the limited cognitive and comprehension abilities of the target population, all questionnaires were completed by their primary caregivers to ensure information accuracy and reliability. Caregivers with multiple children in the target age range (3–12 years) were instructed to answer all questions specifically for the sampled child from the selected class. Electronic questionnaires were distributed through WeChat parent groups in surveyed school classes and administered through Wenjuanxing (Changsha Ranxing Information Technology Co., Ltd., Changsha, China), China’s online survey platform. Prior to the survey, participants were provided with detailed information regarding the study purpose, content, data confidentiality, and their right to voluntary participation through an online informed consent form. Electronic informed consent was obtained before questionnaire completion.

### 2.2. Sample Size Calculation

Based on the sample size calculation formula n = Z^2^α/2p(1−p)/δ^2^, assuming an expected vaccination rate of *p* = 15% [9,10], Z^2^α/2 = 1.96, two-sided α = 0.05, and allowable error of 1.5%, the calculated minimum sample size was 2177. Considering an effective response rate of 90%, at least 2419 questionnaires needed to be distributed. Consequently, this study determined a target sample size of 2500.

### 2.3. Sampling Method

This study employed a multi-stage, stratified, cluster sampling method. Based on economic development level and geographic characteristics within Zhejiang Province, six prefecture-level cities were selected: Hangzhou and Ningbo (sub-provincial cities with high economic development), Jiaxing (northern Zhejiang), Wenzhou (southern Zhejiang), Taizhou (eastern Zhejiang), and Quzhou (western Zhejiang). A total of 10 districts/counties were selected as sample districts/counties according to urban–rural distribution. Within each sample unit, one township/street was randomly selected; from each sample township/street, one primary school and one kindergarten were randomly selected as survey sites. Finally, within each survey site, subjects were randomly selected from kindergartens and primary schools. Prior to formal data collection, a pilot survey was conducted in one primary school and one kindergarten in Ningbo (a non-formal sample site) to test questionnaire procedures and content.

### 2.4. Survey Content

The survey questionnaire was designed based on a review of similar literature [17] and consisted of the following sections. (1) Sociodemographic characteristics: basic information about the child (age, sex, grade, health status, etc.) and caregiver (age, education level, etc.). (2) Characteristics of highly publicized influenza-related death event: caregivers’ awareness of relevant events, information channels, frequency of exposure to such information, and evaluation of the objectivity of media coverage. (3) Previous vaccination history and vaccine hesitancy: including the child’s previous influenza vaccination history, the occurrence of influenza-like illness, previous experiences of vaccination refusal or vaccine hesitancy, and degree of demand for vaccine-related information. (4) Cognition and attitudes: measurement of caregivers’ risk perception of influenza; attitudes toward vaccine effectiveness, safety, and necessity; and vaccination intention. The outcome variable of this study was vaccination intention for the 2025–2026 influenza season, which was dichotomized into two groups for analysis: the willing-to-vaccinate group (including “completely accept” and “accept but will consider”) and the hesitant/refusal group (including “completely refuse,” “refuse but will consider,” and “undecided”). In addition, because 92.02% of caregivers obtained information about the event through social media—with very small sample sizes for other channels—we chose to summarize these variables descriptively rather than including them as separate predictors in inferential models.

### 2.5. Quality Control

To ensure data reliability, the questionnaire design underwent multiple rounds of expert consultation and discussion to establish a robust framework, with concise and unambiguous wording. Prior to formal data collection, a pilot survey was conducted in one primary school and one kindergarten in the Ningbo area to refine ambiguous items and optimize logical sequencing. Before survey implementation, the project team provided comprehensive briefings to all surveyed class teachers, clarifying the survey purpose and completion requirements, with teachers responsible for informing parents and maintaining communication throughout the process. During survey implementation, technical quality control procedures were implemented through Wenjuanxing, including ID number validation, mandatory questions, and logical skip patterns. During data cleaning, dual independent verification was employed, with exclusion criteria including the completion time being deemed too short or too long (less than 2 min or more than 30 min), regular pattern repetition in all responses, and missing key information. ID information was validated for legitimacy, and logical errors in age, sex, and other information were corrected accordingly.

### 2.6. Statistical Analysis

Electronic questionnaire data entry was conducted using Wenjuanxing, and statistical analysis was performed using R software (version 4.5.1). Figures were created using Excel 2019 and Adobe Illustrator CC 2018. Categorical variables were described using frequencies and percentages (%).

Chi-square tests or Fisher’s exact probability tests (when expected frequency < 5) were used to compare distribution differences in characteristics between groups. Univariate logistic regression analysis was used to examine associations between variables and vaccination intention, calculating odds ratios (ORs) and 95% confidence intervals (CIs). Variables with *p* < 0.10 in the univariate analysis were selected as candidates for inclusion in multivariate logistic regression models to identify independent factors associated with vaccination intention. The results were expressed as an adjusted odds ratio (aOR) and 95% confidence interval (CI).

## 3. Results

### 3.1. Characteristics of Study Participants

**Figure 1:**
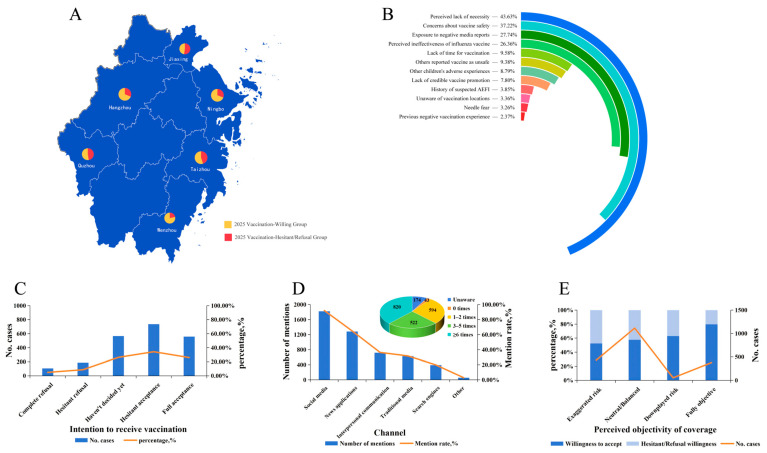
Distribution of influenza vaccination intention and associated factors among caregivers of children in Zhejiang Province. (**A**) Geographic distribution of vaccination intention. (**B**) Primary reasons for vaccination hesitancy or refusal. (**C**) Distribution of intention intensity. (**D**) Information sources and frequency of exposure. (**E**) Association between perceived objectivity of highly publicized influenza event reporting and vaccination intention.

This cross-sectional survey was conducted from March to April 2025. A total of 2210 questionnaires were collected (response rate 88.40%), of which 2153 were considered valid after screening (validity rate 97.42%). The sociodemographic characteristics of the study participants are detailed in Figure 1A and Table 1.

The basic characteristics of the study participants were as follows. Regarding child sex, males (51.79%) slightly outnumbered females (48.21%); regarding child grade distribution, lower primary grades (43.20%) predominated, followed by upper primary grades (36.04%) and kindergarten (20.76%). Household registration type was predominantly urban (49.00%), followed by rural (36.32%) and other (14.68%). Regarding place of residence, Taizhou exhibited the highest proportion (50.72%). The number of children being raised in households was predominantly two (67.16%). Children’s health status in the past year was predominantly rated as “good” (51.09%) and “excellent” (34.42%). Caregiver age was predominantly in the 30–39 years group (62.66%), and caregiver education level was predominantly undergraduate/college (54.90%).

### 3.2. Influenza Vaccination Intention for 2025

Regarding the 2025 influenza season, the distribution of caregivers’ vaccination intention is presented in Figure 1C. Overall, 60.10% (1294/2153) of caregivers expressed acceptance, comprising 25.92% who explicitly indicated “complete acceptance” and 34.18% who indicated they would “accept but still consider.” The hesitant population who indicated they were “not yet decided” accounted for 26.24% (565/2153). The proportion explicitly refusing vaccination (including “completely refuse” and “refuse but still consider”) was 13.66% (294/2153). Vaccination intention for the 2025 influenza season varied across different demographic and characteristic groups (see Table 1 for details).

Among caregivers who explicitly expressed unwillingness or hesitation toward vaccinating their children against influenza (n = 1013), multiple-choice analysis of reasons was conducted. The results demonstrated that caregivers’ primary reasons for refusal or hesitation centered on cognitive aspects regarding vaccine necessity, safety, and efficacy. The three most prevalent reasons were “believing vaccination is unnecessary” (43.63%), “having safety concerns about the vaccine” (37.22%), and “having seen negative media coverage about influenza vaccines” (27.74%). Additionally, “not believing the influenza vaccine is effective” (26.36%) represented another significant barrier factor. Other operational or social reasons accounted for relatively lower proportions.

### 3.3. Awareness and Perception of Highly Publicized Influenza Events

The survey demonstrated that this public health event achieved extremely high awareness among caregivers, reaching 91.92% (1979/2153). Among those aware of the event, information dissemination exhibited high-frequency, social media-dominated characteristics.

Regarding characteristics related to the highly publicized influenza event, 51.79% of caregivers evaluated event coverage as “neutral or balanced,” 20.02% believed it “exaggerated risk,” 17.60% perceived coverage as “completely objective,” 8.08% were “unaware of the event,” and 2.51% believed it “downplayed risk.” Regarding frequency of exposure to event-related coverage, low frequency (0–2 times) accounted for 37.67%, medium frequency (3–5 times) accounted for 24.25%, and high frequency (six or more times) accounted for 38.09%. Social media (WeChat/Weibo/Douyin, etc.) served as the primary source for learning about highly publicized influenza events, with a mention rate of 92.02% (1821/1979), followed by news apps (64.83%); see Figure 1D for detailed information.

Vaccination intention differed substantially across groups with varying objectivity evaluations; see Figure 1E for detailed information. Caregivers who believed coverage was “completely objective” demonstrated the highest vaccination intention, with a willingness-to-vaccinate rate of 79.68% (302/379). Among caregivers who believed coverage was “neutral or balanced,” the willingness-to-vaccinate rate was 57.67% (643/1115). In contrast, among those who believed coverage “exaggerated risk,” the willingness-to-vaccinate rate was only 52.44% (226/431).

### 3.4. Univariate Analysis of Factors Associated with Vaccination Intention

Univariate analysis (using chi-square tests and univariate logistic regression) showed that multiple variables were significantly associated with 2025 influenza vaccination intention (*p* < 0.05). Regarding child grade, caregivers of kindergarten children demonstrated higher vaccination willingness compared to caregivers of lower primary grade children (OR = 1.66, *p* < 0.001). Regarding household registration type, caregivers with other household registration types exhibited higher willingness-to-vaccinate rates than those with urban household registration (OR = 1.33, *p* = 0.04). Regarding place of residence, using Hangzhou as the reference category, Jiaxing (OR = 0.42, *p* < 0.001), Quzhou (OR = 0.50, *p* < 0.001), and Taizhou (OR = 0.56, *p* < 0.001) demonstrated lower willingness-to-vaccinate rates. Regarding the number of children raised in the family, families with two children exhibited lower willingness-to-vaccinate rates compared to families with one child (OR = 0.73, *p* < 0.001). The “poor” child health status group demonstrated significantly lower willingness-to-vaccinate rates than the “excellent” group (OR = 0.25, *p* = 0.02). Regarding event-related characteristics, caregivers who believed coverage was “completely objective” displayed higher willingness-to-vaccinate rates (OR = 3.75, *p* < 0.001), and the high exposure frequency group exhibited higher willingness-to-vaccinate rates (OR = 1.70, *p* < 0.001). Regarding previous vaccination history, caregivers whose children were not vaccinated against influenza in the 2024 season demonstrated lower willingness-to-vaccinate rates (OR = 0.19, *p* < 0.001), and caregivers whose children did not have influenza-like illness in 2024 exhibited lower willingness-to-vaccinate rates (OR = 0.71, *p* < 0.001). Caregivers with no history of vaccination refusal (OR = 7.29, *p* < 0.001) or vaccine hesitancy (OR = 5.16, *p* < 0.001) demonstrated higher willingness-to-vaccinate rates. Regarding cognition and attitude variables, risk perception (not believing influenza is the common cold, not believing influenza resolves within 3–7 days), vaccine attitudes (such as believing in vaccine effectiveness, believing vaccines are important), and vaccination intention (following physician recommendations to vaccinate children against influenza) were all associated with higher vaccination intention (all *p* < 0.05). See Table 1 for details.

### 3.5. Multivariate Analysis of Factors Associated with Vaccination Intention

Multivariate logistic regression analysis demonstrated that multiple variables were significantly associated with 2025 influenza vaccination intention (*p* < 0.05). Regarding place of residence, compared with Hangzhou, Jiaxing (aOR = 0.33, *p* < 0.001), Quzhou (aOR = 0.52, *p* = 0.03), and Taizhou (aOR = 0.57, *p* < 0.001) demonstrated lower caregiver willingness-to-vaccinate rates. The “poor” child health status group exhibited lower willingness-to-vaccinate rates (aOR = 0.19, *p* = 0.02). Regarding previous vaccination history, children not vaccinated against influenza in 2024 demonstrated lower willingness-to-vaccinate rates (aOR = 0.41, *p* < 0.001), and children who experienced influenza-like illness in 2024 exhibited higher willingness-to-vaccinate rates (aOR = 1.36, *p* = 0.02). Caregivers without a history of vaccination refusal demonstrated higher willingness-to-vaccinate rates (aOR = 2.74, *p* < 0.001), and caregivers without a history of vaccine hesitancy also exhibited higher willingness-to-vaccinate rates (aOR = 1.47, *p* < 0.001). Caregivers who required more vaccine information demonstrated higher willingness-to-vaccinate rates, with “needed” (aOR = 6.42, *p* < 0.001) and “very much needed” (aOR = 8.83, *p* < 0.001) groups showing substantially higher rates than the “very much not needed” group. Regarding cognition and attitudes, caregivers who did not believe influenza symptoms resolve within 3 to 7 days demonstrated higher willingness-to-vaccinate rates (aOR = 1.39, *p* = 0.01), caregivers who did not believe in vaccine protective effects exhibited lower willingness-to-vaccinate rates (aOR = 0.60, *p* < 0.001), and caregivers who did not believe most parents would have their children vaccinated demonstrated lower willingness-to-vaccinate rates (aOR = 0.70, *p* = 0.01). Other variables were no longer statistically significant in multivariate analysis. See Table 1 for details.

## 4. Discussion

This study comprehensively examined the current status of childhood influenza vaccination intention and its influencing factors in the context of a highly publicized influenza event. Through a cross-sectional survey of 2153 caregivers in Zhejiang Province, we found that, in the aftermath of the highly publicized influenza event, the overall vaccination intention among caregivers of children reached 60.10%; however, a substantial proportion of caregivers remained hesitant or refused vaccination. Multivariate analysis revealed that caregivers’ media cognition (such as the evaluation of coverage objectivity), previous vaccination behavior, beliefs regarding vaccine protective effects, and information needs represented independent key factors influencing their current vaccination decisions, whereas the influence of certain traditional demographic variables was no longer statistically significant in multivariate analysis. This finding underscores the role of cognitive–psychological and behavioral experience factors in shaping health decision-making in the context of sudden public health events.

Media coverage and dissemination of the highly publicized influenza event demonstrated an association with caregivers’ vaccination intention. An exceptionally high awareness rate of 91.92% was observed, with social media identified as the predominant information source (92.02%). Research conducted as early as 2018 demonstrated that social media had become the dominant channel for vaccine-related information dissemination. For instance, Han et al. reported that 86.8% of respondents had heard of the vaccine scandal, with social media being the most common initial channel [18]. These findings underscore the substantial communicative reach and societal influence of such events in the digital media era.

Further analysis revealed that caregivers’ evaluation of media coverage objectivity was associated with vaccination intention. Caregivers who believed coverage was “completely objective” exhibited higher vaccination intention compared to those who believed coverage “exaggerated risk.” This finding indicates that information dissemination about highly publicized influenza events through the media may influence health behaviors (whether to vaccinate children against influenza) by affecting public risk perception (the perceived danger of influenza) and event attribution (the reasons underlying publicized influenza events). When caregivers believe that coverage objectively presents the severity of influenza, they may be more inclined to view it as a public health threat warranting serious attention, thereby strengthening preventive motivation. Conversely, when media outlets deliberately exaggerate risks, adverse consequences may arise. For instance, during the 2018 Changchun Biolabs vaccine incident, although no cases of death or other severe consequences relevant to the substandard rabies and DPT vaccines have been documented, the substandard vaccines were reported as being poisonous on social media [19]. This type of factually unsupported negative coverage substantially amplified public panic and eroded trust, leading to a decline in vaccine confidence among the majority of guardians. As demonstrated in the study by Wang X et al., over 85% of guardians developed skepticism toward vaccination [20]. Therefore, in the aftermath of similar high-profile incidents, authoritative media should adopt timely, scientifically rigorous, and comprehensive pro-vaccine communication strategies to enhance public acceptance of relevant vaccines, thereby maximizing their potential to promote positive health behaviors [21].

Previous vaccination behavior represents one of the key predictors of future vaccination intention. Children who received influenza vaccination during the previous season (2024) demonstrated a positive association with vaccination intention in the current season (2025) (aOR = 0.41, *p* < 0.001), supporting the “inertia” of vaccination behavior [22,23,24]. Simultaneously, experience of influenza or influenza-like illness during the previous year was also associated with higher vaccination intention (aOR = 1.36, *p* = 0.02). Thus, individuals’ direct behavioral experience proves instrumental in shaping subsequent decisions. Positive vaccination experience solidifies healthy behavior patterns, while negative illness experience may transform into personal cognition of disease risk, with both working together to effectively shape and consolidate caregivers’ health beliefs and subsequent behavioral intentions. Notably, over 70% (72.55%) of children were not vaccinated in the 2024 season, and this population constitutes the predominant body of currently hesitant and refusing caregivers. Therefore, the critical pathway to improving overall vaccination rates may lie in conducting targeted interventions directed toward the majority of families whose children were not vaccinated during the previous influenza season.

Multivariate analysis demonstrated that caregivers who did not believe in the protective effect of influenza vaccines (aOR = 0.60, *p* < 0.001) and those who did not agree that “most parents would have their children vaccinated” (aOR = 0.70, *p* = 0.01) exhibited significantly lower vaccination intention. Similarly, studies conducted by Amicizia, D. and A.M. Hofstetter et al. corroborated analogous findings [24,25]. Additionally, the belief that influenza cannot resolve spontaneously within a short timeframe (aOR = 1.39, *p* = 0.01) was associated with higher vaccination intention, reflecting caregivers’ risk perception regarding disease severity and susceptibility. Specifically, caregivers’ recognition of the severity of influenza demonstrated a positive correlation with higher acceptance of influenza vaccination [26]. Furthermore, as information need increased, vaccination intention correspondingly increased: compared with caregivers who believed they “very much did not need” additional vaccine information, those who indicated they “needed” (aOR = 6.42, *p* < 0.001) and “very much needed” (aOR = 8.83, *p* < 0.001) information demonstrated several-fold higher vaccination intention. This finding also appears somewhat counterintuitive, as greater information need might initially be expected to reflect uncertainty or hesitancy rather than stronger vaccination intention. One possible explanation is that caregivers who actively acknowledge a need for vaccine information may already be experiencing an internal cue to action; that is, recognizing an information gap itself may motivate them to seek knowledge and ultimately strengthen their intention to vaccinate [27]. This interpretation is also supported by previous research showing that insufficient information was one of the most commonly reported reasons among caregivers with low vaccination intention [28]. Taken together, these findings suggest that information may play a dual role: lack of information may contribute to low vaccination intention, whereas an awareness of that lack and active information seeking may function as triggers that promote stronger intention. This also suggests that current vaccine hesitancy among some caregivers may not necessarily stem from entrenched refusal attitudes but rather from information asymmetry or knowledge gaps, which may represent an important opportunity for targeted communication interventions.

In addition, the finding that weaker vaccination intention was significantly associated with poorer child health status (aOR = 0.19, 95% CI: 0.05–0.76) appears somewhat counterintuitive, as caregivers of children who may benefit most from influenza vaccination might be expected to show stronger willingness. One possible explanation is that caregivers of children with poorer health status may have greater concerns about vaccine safety, potential adverse reactions, or the child’s ability to tolerate vaccination. Similar patterns have been reported in previous studies on COVID-19 vaccination. One study found lower vaccination intention among caregivers of children with chronic illness [29], while another reported that caregivers of children with asthma, medical complexity, or mental illness were not more willing to vaccinate their children [30]. These findings suggest that clinicians and public health practitioners should not assume that families of children with poorer health status are already motivated to vaccinate; instead, they may require more proactive communication and reassurance regarding the safety and importance of influenza vaccination, particularly for children at higher risk of severe outcomes.

Regarding the translation of vaccination intention into actual vaccination behavior, this study only assessed caregivers’ vaccination intention rather than real-world vaccination uptake. Nevertheless, accumulating evidence has verified a strong positive linkage between intention and subsequent behavior. Prior research focusing on COVID-19 has shown strong intention-to-behavior conversion: 72% of children whose caregivers intended COVID-19 vaccination were vaccinated, compared with only 28% when caregivers were unsure or did not intend [30]. Similarly, another study on childhood seasonal influenza vaccination in Singapore found that each one-unit increase in willingness to vaccinate was associated with a 1.58-fold increase in actual vaccination (PR = 1.58, 95% CI: 1.24–2.04) [31]. These findings confirm that vaccination intention is a reliable predictor of real vaccination behavior. Therefore, exploring the influencing factors of vaccination intention and conducting targeted intervention are effective approaches to improving childhood influenza vaccination coverage in practice.

## 5. Limitations

This study has several limitations. First, the cross-sectional design cannot infer causality, only associations. Second, although the sample size was substantial, uneven regional distribution may affect the representativeness of inferences regarding the entire province. Third, recruiting caregivers through WeChat parent groups may have introduced selection bias by overrepresenting more engaged, health-literate, or digitally active individuals. Fourth, the survey was conducted shortly after the event occurred, and vaccination intention had not yet fully translated into actual vaccination behavior, and intention may change over time, with long-term effects requiring follow-up.

## 6. Conclusions

Through this comprehensive cross-sectional survey, we systematically evaluated influenza vaccination intention and its key influencing factors among caregivers of children in Zhejiang Province in the context of a highly publicized influenza event. The results demonstrated that the overall vaccination intention among caregivers of children in the aftermath of the event reached 60.10%; however, caregivers’ decisions were predominantly driven by media cognition (such as evaluation of coverage objectivity), previous vaccination behavior, beliefs regarding vaccine protective effects, and information needs, rather than traditional demographic characteristics. This finding reveals the dominant role of cognitive–psychological and behavioral experience factors in health decision-making during sudden public health events. Future efforts should prioritize targeted information dissemination to caregivers with low perceived information need (a paradoxical high-risk group) and leverage prior vaccination behavior as an entry point for intervention. Longitudinal and intervention studies are needed to confirm whether information needs translate into actual uptake and whether improving media cognition and vaccine beliefs can effectively reduce hesitancy. Moreover, translating stated intention into action will require healthcare providers to proactively discuss vaccine protective effects and address information deficits, particularly among caregivers with no need for vaccine information.

## Figures and Tables

**Table 1:** Influenza vaccination intention and associated factors among caregivers of children in Zhejiang Province during the 2024–2025 surveillance season.

Variable	Total (n = 2153)		2025 Hesitant/Refusal Group	2025 Willing Group	χ^2^		Univariate Analysis		Multivariable Analysis	
					Statistic	*p*-Value	OR (95% CI)	*p*-Value	aOR (95% CI)	*p*-Value
I. Sociodemographic Characteristics										
Child sex										
Male		1115 (51.79%)	436 (39.10%)	679 (60.90%)	0.5423	0.4615	1.00		-	
Female		1038 (48.21%)	423 (40.75%)	615 (59.25%)			0.93 (0.79, 1.11)	0.44	-	
Child educational level										
Lower primary (Grades 1–3)		930 (43.20%)	382 (41.08%)	548 (58.92%)	27.3062	<0.001	1.00			
Upper primary (Grades 4–6)		776 (36.04%)	345 (44.46%)	431 (55.54%)			0.87 (0.72, 1.06)	0.16	0.82 (0.62, 1.07)	0.14
Kindergarten		447 (20.76%)	132 (29.53%)	315 (70.47%)			1.66 (1.31, 2.12)	<0.001	0.88 (0.57, 1.34)	0.55
Household registration type										
Urban local district/county		1055 (49.00%)	423 (40.09%)	632 (59.91%)	7.0643	0.0292	1.00			
Rural local district/county		782 (36.32%)	330 (42.20%)	452 (57.80%)			0.92 (0.76, 1.11)	0.36	1 (0.75, 1.34)	1
Other		316 (14.68%)	106 (33.54%)	210 (66.46%)			1.33 (1.02, 1.73)	0.04	1.07 (0.68, 1.7)	0.76
Residence location										
Hangzhou		452 (20.99%)	141 (31.19%)	311 (68.81%)	57.106	<0.001	1.00			
Jiaxing		115 (5.34%)	60 (52.17%)	55 (47.83%)			0.42 (0.27, 0.63)	<0.001	0.33 (0.18, 0.6)	0
Ningbo		270 (12.54%)	83 (30.74%)	187 (69.26%)			1.02 (0.74, 1.42)	0.90	0.7 (0.41, 1.2)	0.2
Quzhou		141 (6.55%)	67 (47.52%)	74 (52.48%)			0.50 (0.34, 0.74)	<0.001	0.52 (0.29, 0.93)	0.03
Taizhou		1092 (50.72%)	490 (44.87%)	602 (55.13%)			0.56 (0.44, 0.70)	<0.001	0.57 (0.39, 0.84)	0
Wenzhou		83 (3.86%)	18 (21.69%)	65 (78.31%)			1.64 (0.95, 2.94)	0.08	0.8 (0.37, 1.79)	0.58
Number of children in household										
1		572 (26.57%)	198 (34.62%)	374 (65.38%)	9.4645	0.0088	1.00			
2		1446 (67.16%)	608 (42.05%)	838 (57.95%)			0.73 (0.60, 0.89)	<0.001	1.01 (0.77, 1.33)	0.94
≥3		135 (6.27%)	53 (39.26%)	82 (60.74%)			0.82 (0.56, 1.21)	0.31	1.03 (0.62, 1.72)	0.91
Health status during past year										
Excellent		741 (34.42%)	302 (40.76%)	439 (59.24%)	*	0.0555	1.00			
Good		1100 (51.09%)	429 (39.00%)	671 (61.00%)			1.08 (0.89, 1.30)	0.45	0.98 (0.75, 1.28)	0.9
Fair		297 (13.79%)	117 (39.39%)	180 (60.61%)			1.06 (0.80, 1.40)	0.69	0.83 (0.57, 1.22)	0.34
Poor		15 (0.70%)	11 (73.33%)	4 (26.67%)			0.25 (0.07, 0.74)	0.02	0.19 (0.04, 0.74)	0.02
Age range of guardians										
≥50		63 (2.93%)	29 (46.03%)	34 (53.97%)	3.518	0.3184	1.00	0.15	-	
40–49		594 (27.59%)	249 (41.92%)	345 (58.08%)			1.18 (0.70, 1.99)	0.53	-	
30–39		1349 (62.66%)	529 (39.21%)	820 (60.79%)			1.32 (0.79, 2.19)	0.28	-	
20–29		147 (6.83%)	52 (35.37%)	95 (64.63%)			1.56 (0.85, 2.84)	0.15	-	
Guardian’s highest educational attainment										
Junior high school or below		467 (21.69%)	178 (38.12%)	289 (61.88%)	3.0444	0.3848	1.00		-	
High school/vocational school		432 (20.07%)	175 (40.51%)	257 (59.49%)			0.90 (0.69, 1.18)	0.46	-	
College/undergraduate		1182 (54.90%)	483 (40.86%)	699 (59.14%)			0.89 (0.71, 1.11)	0.31	-	
Postgraduate or above		72 (3.34%)	23 (31.94%)	49 (68.06%)			1.31 (0.78, 2.26)	0.31	-	
II. Highly Publicized Influenza Events Characteristics										
Perceived objectivity of media reporting										
Unaware of the incident	174 (8.08%)		85 (48.85%)	89 (51.15%)	79.9185	<0.001	1.00			
Exaggerated risk	431 (20.02%)		205 (47.56%)	226 (52.44%)			1.05 (0.74, 1.50)	0.77	0.98 (0.58, 1.64)	0.92
Neutral or balanced	1115 (51.79%)		472 (42.33%)	643 (57.67%)			1.30 (0.94, 1.79)	0.11	0.94 (0.59, 1.52)	0.81
Somewhat downplayed risk	54 (2.51%)		20 (37.04%)	34 (62.96%)			1.62 (0.87, 3.08)	0.13	1.08 (0.47, 2.48)	0.86
Completely objective	379 (17.60%)		77 (20.32%)	302 (79.68%)			3.75 (2.54, 5.54)	<0.001	1.35 (0.77, 2.39)	0.3
Frequency of exposure to incident reporting										
Low (0–2 times)	811 (37.67%)		378 (46.61%)	433 (53.39%)	27.7465	<0.001	1.00			
Moderate (3–5 times)	522 (24.25%)		203 (38.89%)	319 (61.11%)			1.37 (1.10, 1.72)	0.01	1.02 (0.75, 1.4)	0.88
High (≥6 times)	820 (38.09%)		278 (33.90%)	542 (66.10%)			1.70 (1.39, 2.08)	<0.001	1.22 (0.91, 1.63)	0.18
III. Vaccination History and Vaccine Hesitancy										
Has your child received influenza vaccine during the 2024 influenza season?										
Yes	591 (27.45%)		92 (15.57%)	499 (84.43%)	199.7123	<0.001	1.00			
No	1562 (72.55%)		767 (49.10%)	795 (50.90%)			0.19 (0.15, 0.24)	<0.001	0.41 (0.3, 0.55)	<0.001
Did your child have influenza or experience fever ≥38 °C with cough or sore throat during the 2024 influenza season?										
Yes	861 (39.99%)		302 (35.08%)	559 (64.92%)	13.5811	0.0002				
No	1292 (60.01%)		557 (43.11%)	735 (56.89%)			0.71(0.60, 0.85)	<0.001	0.73(0.56,0.95)	0.02
Have you ever refused influenza vaccination for this child?										
Yes	554 (25.73%)		410 (74.01%)	144 (25.99%)	360.0086	<0.001	1.00			
No	1599 (74.27%)		449 (28.08%)	1150 (71.92%)			7.29 (5.87, 9.10)	<0.001	2.74 (2.03, 3.72)	<0.001
Have you ever been unwilling or hesitant to have this child vaccinated against influenza?										
Yes	954 (44.31%)		581 (60.90%)	373 (39.10%)	313.5836	<0.001	1.00			
No	1199 (55.69%)		278 (23.19%)	921 (76.81%)			5.16 (4.29, 6.23)	<0.001	1.47 (1.13, 1.92)	<0.001
Did you access information about influenza vaccination through media channels during the past year?										
Yes	1679 (77.98%)		649 (38.65%)	1030 (61.35%)	4.6878	0.0304	1.00			
No	474 (22.02%)		210 (44.30%)	264 (55.70%)			0.79 (0.64, 0.97)	0.03	1.20 (0.91, 1.6)	0.20
Do you feel the need to obtain more information about influenza vaccines and vaccination?										
Not needed at all	62 (2.88%)		46 (74.19%)	16 (25.81%)	406.7801	<0.001	1.00			
Not needed	197 (9.15%)		146 (74.11%)	51 (25.89%)			1.00 (0.53, 1.97)	0.99	1.75 (0.7, 4.48)	0.24
Neutral	567 (26.34%)		353 (62.26%)	214 (37.74%)			1.74 (0.98, 3.25)	0.07	2.92 (1.26, 6.97)	0.01
Needed	926 (43.01%)		252 (27.21%)	674 (72.79%)			7.69 (4.37, 14.23)	<0.001	6.42 (2.79, 15.21)	<0.001
Highly needed	401 (18.63%)		62 (15.46%)	339 (84.54%)			15.72 (8.54, 30.30)	<0.001	8.83 (3.71, 21.59)	<0.001
IV. Knowledge and Attitudes										
(A) Risk Perception										
Do you believe influenza is the same as the common cold?										
Yes	316 (14.68%)		153 (48.42%)	163 (51.58%)	10.7986	0.001	1.00			
No	1837 (85.32%)		706 (38.43%)	1131 (61.57%)			1.50 (1.18, 1.91)	<0.001	1.13 (0.8, 1.58)	0.49
Do you believe symptoms of typical influenza usually resolve spontaneously within 3–7 days?										
Yes	996 (46.26%)		464 (46.59%)	532 (53.41%)	34.0603	<0.001	1.00			
No	1157 (53.74%)		395 (34.14%)	762 (65.86%)			1.68 (1.41, 2.00)	<0.001	1.39 (1.08, 1.77)	0.01
(B) Vaccine Attitudes										
Do you trust that influenza vaccine can protect your child from severe illness?										
Yes	1481 (68.79%)		437 (29.51%)	1044 (70.49%)	212.2538	<0.001	1.00			
No	672 (31.21%)		422 (62.80%)	250 (37.20%)			0.25 (0.20, 0.30)	<0.001	0.60 (0.46, 0.79)	<0.001
Most parents have their children vaccinated.										
Yes	1567 (72.78%)		478 (30.50%)	1089 (69.50%)	210.422	<0.001	1.00			
No	586 (27.22%)		381 (65.02%)	205 (34.98%)			0.24 (0.19, 0.29)	<0.001	0.70 (0.53, 0.93)	0.01
Influenza vaccination is important for my child’s health.										
Strongly disagree	55 (2.55%)		29 (52.73%)	26 (47.27%)	474.2299	<0.001	1.00			
Disagree	54 (2.51%)		44 (81.48%)	10 (18.52%)			0.25 (0.10, 0.59)	<0.001	0.80 (0.14, 4.28)	0.80
Neutral	703 (32.65%)		478 (67.99%)	225 (32.01%)			0.53 (0.30, 0.92)	0.02	0.69 (0.15, 2.81)	0.62
Agree	813 (37.76%)		242 (29.77%)	571 (70.23%)			2.63 (1.52, 4.59)	<0.001	0.80 (0.17, 3.24)	0.77
Strongly agree	528 (24.52%)		66 (12.50%)	462 (87.50%)			7.81 (4.34, 14.15)	<0.001	0.95 (0.2, 3.95)	0.94
Influenza vaccines are effective.										
Strongly disagree	56 (2.60%)		32 (57.14%)	24 (42.86%)	492.5616	<0.001	1.00			
Disagree	62 (2.88%)		54 (87.10%)	8 (12.90%)			0.20 (0.08, 0.47)	0.00	0.51 (0.09, 2.95)	0.45
Neutral	732 (34.00%)		485 (66.26%)	247 (33.74%)			0.68 (0.39, 1.19)	0.17	1.01 (0.22, 4.83)	0.99
Agree	873 (40.55%)		248 (28.41%)	625 (71.59%)			3.36 (1.95, 5.87)	<0.001	1.53 (0.33, 7.37)	0.59
Strongly agree	430 (19.97%)		40 (9.30%)	390 (90.70%)			13.00 (7.03, 24.46)	<0.001	2.24 (0.45, 11.43)	0.32
I believe information about influenza vaccines is reliable and trustworthy.										
Strongly disagree	53 (2.46%)		32 (60.38%)	21 (39.62%)	306.3426	<0.001	1.00			
Disagree	101 (4.69%)		65 (64.36%)	36 (35.64%)			0.84 (0.43, 1.68)	0.63	0.97 (0.25, 3.69)	0.96
Neutral	912 (42.36%)		519 (56.91%)	393 (43.09%)			1.15 (0.66, 2.06)	0.62	1.26 (0.35, 4.45)	0.72
Agree	771 (35.81%)		207 (26.85%)	564 (73.15%)			4.15 (2.36, 7.46)	<0.001	1.66 (0.46, 6.02)	0.44
Strongly agree	316 (14.68%)		36 (11.39%)	280 (88.61%)			11.85 (6.24, 23.04)	<0.001	0.92 (0.22, 3.84)	0.91
Vaccination is a good way to protect my child from illness.										
Strongly disagree	87 (4.04%)		55 (63.22%)	32 (36.78%)	293.7472	<0.001	1.00			
Disagree	199 (9.24%)		117 (58.79%)	82 (41.21%)			1.20 (0.72, 2.04)	0.48	1.91 (0.73, 5.16)	0.19
Neutral	870 (40.41%)		475 (54.60%)	395 (45.40%)			1.43 (0.91, 2.28)	0.12	1.69 (0.69, 4.3)	0.26
Agree	696 (32.33%)		181 (26.01%)	515 (73.99%)			4.89 (3.08, 7.88)	<0.001	1.31 (0.52, 3.38)	0.57
Strongly agree	301 (13.98%)		31 (10.30%)	270 (89.70%)			14.97 (8.54, 26.91)	<0.001	1.11 (0.35, 3.48)	0.86
(C) Vaccination Intention										
Under normal circumstances, I would follow my doctor or healthcare provider’s recommendation to have my child vaccinated against influenza.										
Strongly disagree	47 (2.18%)		29 (61.70%)	18 (38.30%)	533.1262	<0.001	1.00			
Disagree	70 (3.25%)		61 (87.14%)	9 (12.86%)			0.24 (0.09, 0.58)	<0.001	0.25 (0.04, 1.33)	0.11
Neutral	627 (29.12%)		443 (70.65%)	184 (29.35%)			0.67 (0.37, 1.26)	0.20	0.42 (0.09, 1.98)	0.28
Agree	1010 (46.91%)		290 (28.71%)	720 (71.29%)			4.00 (2.21, 7.44)	<0.001	0.88 (0.19, 4.06)	0.87
Strongly agree	399 (18.53%)		36 (9.02%)	363 (90.98%)			16.25 (8.32, 32.64)	<0.001	2.46 (0.48, 12.78)	0.28

- Indicates variable not included in multivariate analysis. * Indicates Fisher’s exact test was used.

## Data Availability

Data are contained within the article.
